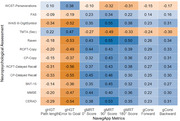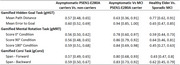# Spatial cognition as an early cognitive Marker for Alzheimer's disease: evidence from NavegApp, an innovative serious game for cognitive assessment

**DOI:** 10.1002/alz70856_104900

**Published:** 2026-01-08

**Authors:** Juan Pablo Sanchez, Diego Camilo Díaz, Stella Maris Valencia, David Fernando Aguillón Niño, Mauricio Garcia‐Barrera, Daniel C. Aguirre‐Acevedo, Natalia Trujillo Orrego

**Affiliations:** ^1^ Grupo de Neurociencias de Antioquia, Facultad de Medicina, Universidad de Antioquia, Medellín, Antioquia, Colombia; ^2^ University of Antioquia, Medellin, Colombia; ^3^ Fundación Universitaria Luis Amigó, Medellín, Antioquia, Colombia; ^4^ Grupo de investigación en salud mental, Universidad de Antioquia, Medellin, Antioquia, Colombia; ^5^ Grupo de Neurociencias de Antioquia, Universidad de Antioquia, Medellín, Antioquia, Colombia; ^6^ University of Victoria, Victoria BC, Canada; ^7^ Grupo de Neurociencias de Antioquia, Medellín, Antioquia, Colombia; ^8^ Atlantic Fellow for Equity in Brain Health at University of California, San Francisco / Trinity College of Dublin, San Francisco, CA, USA; ^9^ Florida International University, Miami, FL, USA

## Abstract

**Background:**

Limited access to diagnostic technologies in low and middle‐income countries hinders the early detection of Alzheimer's Disease (AD). Advances in digital neuropsychology have enabled the development of tools like NavegApp, a serious game‐based platform that evaluates allocentric navigation, mental rotation, and visuospatial memory—domains affected during the preclinical and prodromal stages of AD. While previous studies have confirmed NavegApp's usability and content validity, further evidence of its construct validity and diagnostic accuracy is required to support its potential implementation in primary care settings. The analysis of cognitive changes during these early disease stages, particularly in individuals carrying causative mutations for early‐onset familial AD, is essential to advance the clinical utility of innovative digital cognitive markers. Therefore, the main objective of this study was to evaluate the construct validity and diagnostic accuracy of NavegApp's spatial cognition metrics across the AD spectrum.

**Method:**

A retrospective observational study was conducted with 226 participants classified into five groups, including presymptomatic and symptomatic PSEN1‐E280A carriers. Construct validity was assessed by examining correlations between NavegApp metrics and standard neuropsychological assessments. Group performance differences were analyzed using effect size estimates, controlling for sex, education, and age, while diagnostic accuracy was evaluated using ROC curve analysis.

**Result:**

NavegApp's spatial cognition metrics demonstrated moderate associations with general cognitive status, memory, and visuospatial domains. Diagnostic accuracy analysis revealed excellent discriminative capacity for identifying symptomatic PSEN1‐E280A carriers compared to asymptomatic participants, particularly in allocentric navigation (AUC‐ROC = 0.94–0.97). However, diagnostic performance for early preclinical detection was limited (AUC‐ROC = 0.66). In contrast, metrics showed acceptable accuracy for distinguishing sporadic MCI from healthy elder controls (AUC‐ROC = 0.77).

**Conclusion:**

The findings demonstrate the feasibility of NavegApp as an innovative digital tool with potential applications in cognitive screening, particularly in underserved populations. Further research is needed to validate its clinical utility in broader settings and to explore the variability of cognitive changes across the AD spectrum. This evidence could inform its integration into clinical workflows for the preclinical and prodromal detection of AD.